# Environmental behaviour of iron and steel slags in coastal settings

**DOI:** 10.1007/s11356-024-33897-4

**Published:** 2024-06-14

**Authors:** Alex L. Riley, James Cameron, Ian T. Burke, Patrizia Onnis, John M. MacDonald, Catherine J. Gandy, Richard A. Crane, Patrick Byrne, Sean Comber, Adam P. Jarvis, Karen A. Hudson-Edwards, William M. Mayes

**Affiliations:** 1 School of Environmental Sciences, University of Hull, Kingston upon Hull, UK; 2 School of Earth and Environment, University of Leeds, Leeds, UK; 3 Environment and Sustainability Institute and Camborne School of Mines, University of Exeter, Penryn, UK; 4 School of Geographical and Earth Sciences, University of Glasgow, Glasgow, UK; 5 School of Engineering, Newcastle University, Newcastle upon Tyne, UK; 6 School of Biological and Environmental Sciences, Liverpool John Moores University, Liverpool, UK; 7 School of Geography, Earth and Environmental Sciences, University of Plymouth, Plymouth, UK

**Keywords:** Iron and steel slag, Legacy waste, Leachate formation, Coastal pollution, Synchrotron, Waste geochemistry

## Abstract

Iron and steel slags have a long history of both disposal and beneficial use in the coastal zone. Despite the large volumes of slag deposited, comprehensive assessments of potential risks associated with metal(loid) leaching from iron and steel by-products are rare for coastal systems. This study provides a national-scale overview of the 14 known slag deposits in the coastal environment of Great Britain (those within 100 m of the mean high-water mark), comprising geochemical characterisation and leaching test data (using both low and high ionic strength waters) to assess potential leaching risks. The seaward facing length of slag deposits totalled at least 76 km, and are predominantly composed of blast furnace (iron-making) slags from the early to mid-20th Century. Some of these form tidal barriers and formal coastal defence structures, but larger deposits are associated with historical coastal disposal in many former areas of iron and steel production, notably the Cumbrian coast of England. Slag deposits are dominated by melilite phases (e.g. gehlenite), with evidence of secondary mineral formation (e.g. gypsum, calcite) indicative of weathering. Leaching tests typically show lower element (e.g. Ba, V, Cr, Fe) release under seawater leaching scenarios compared to deionised water, largely ascribable to the pH buffering provided by the former. Only Mn and Mo showed elevated leaching concentrations in seawater treatments, though at modest levels (<3 mg/L and 0.01 mg/L, respectively). No significant leaching of potentially ecotoxic elements such as Cr and V (mean leachate concentrations <0.006 mg/L for both) were apparent in seawater, which micro-X-Ray Absorption Near Edge Structure (μXANES) analysis show are both present in slags in low valence (and low toxicity) forms. Although there may be physical hazards posed by extensive erosion of deposits in high-energy coastlines, the data suggest seawater leaching of coastal iron and steel slags in the UK is likely to pose minimal environmental risk.

**Supplementary Information:**

The online version contains supplementary material available at 10.1007/s11356-024-33897-4.

## Introduction

Blast furnace (BF) and basic oxygen furnace (BOF) slags are by-products of iron and steel production with an annual global production in the region of 400 million tonnes (World Steel Association 2021). The re-use of iron and steel making slags has long been practised in construction (e.g. aggregate use and cement binder substitutes) (Santamaría et al. 2023; Rasmus et al. 2023; Lee 1974) and an emerging range of environmental applications such as a filter media, soil amendment, and for atmospheric carbon capture (Gomes et al. 2021). Despite this breadth of potential after-uses, slag production has historically outstripped reuse and as such disposal of slags in terrestrial and coastal settings has been commonplace.

Iron and steel-making slags are typically comprised of a range of Ca (alumino-)silicate and oxide minerals, with some Fe oxides (in BOF slag) and trace element enrichment (notably Mn, Cr and V: Proctor et al. 2000; Piatak et al. 2019, Piatak et al. 2015). Upon weathering, these minerals can generate alkaline leachates dominated by Ca-OH type waters. In terrestrial settings, such leachates can be characterised by high pH (>12), metal(loid) enrichment, high rates of secondary carbonate formation and precipitation, and have been documented to be of ecological concern downstream of slag disposal sites (e.g. Koryak et al. 1998; Hull et al. 2014). Metal(loid) enrichment in leaching products includes oxyanion-forming elements soluble at high pH such as V and Mo, alongside alkaline earth metals such as Ba that can reach concentrations of environmental concern (Hobson et al. 2018; Matern et al. 2013; Fӓllman 2000). A range of management options have been developed to address these concerns in freshwater settings such as leachate dosing (Gomes et al. 2018), carbon dioxide sparging (Roadcap et al. 2006) and the use of constructed wetlands (Gomes et al. 2019) for pH neutralisation and trace element removal.

The environmental behaviour of iron and steel making slags have generally received less attention in estuarine and marine settings than in terrestrial and freshwater environments. Iron and steel by-products have been disposed of in large quantities in coastal margins given the coastal location of many steel mills (for water use, raw material import and product export), and for land reclamation purposes in jurisdictions with land scarcity pressures (Ding et al. 2019). Furthermore, there are a range of established and emerging environmental applications for slags in coastal restoration schemes, such as a substrate for coral rehabilitation and seagrass restoration in Egypt (Mohammed et al. 2012), South Korea (Park et al. 2022) and Japan (Nishijima et al. 2015; Okuda et al. 2014); use of slag in aquaculture or as a broader marine fertiliser in areas of Fe deficiency (Sakurai et al. 2020), and long-standing uses in coastal defence structures (Lee 1974; Foekema et al. 2021).

As such, some studies have sought to characterise long-term leaching from slags in coastal settings. Han et al. (2019) demonstrated preferential leaching of Fe and Mn from BOF slags under elevated salinity. In long term leaching experiments, Fe and Mn were released consistently from the slag at relatively modest concentrations (average of 0.03 and 0.003 mg/L respectively) and suggested there would unlikely to be any ecotoxicological consequence on receiving coastal systems (Han et al. 2019). This low-level leaching of elements like Fe has even been deemed a potentially positive influence on marine systems that are Fe-limited (e.g. where nutrient input from rivers is diminished by river regulation and landslide control: Sakurai et al. 2020). However, most studies often overlook assessment of potentially hazardous species such as V, Cr and Mo which have been demonstrated to be mobile in leaching products in freshwater settings at levels of potential environmental concern (Hobson et al. 2017; Matern et al. 2013). Research by Foekema et al. (2021) has suggested that V is a good low concentration tracer of leaching products from BOF slag in coastal settings, suggesting some mobility of these potentially hazardous species. However, information on the potential environmental risks of coastal disposal and reuse of iron and steel making slags is currently limited.

The UK has extensive deposits of iron and steel-making slags that have been historically deposited in the coastal zone (Riley et al. 2020). This was driven by the coastal location of many iron and steel making areas given the geographic advantages for material imports and export, proximity to water supply, and what was historically perceived as a low land value for coastal areas subject to inundation. The growing interest in reworking legacy slag deposits for value recovery (notably for aggregate and low carbon cement production) could also increase risk of pollutant release (Piatak 2018; Riley et al. 2020). As such, the UK provides a useful case for assessing potential environmental risks of slags that have been emplaced in a range of coastal settings. This paper aims to (1) quantify the extent and nature of coastal iron and steel slag deposits in Great Britain, (2) assess the composition of legacy iron and steel slags at a range of coastal sites, and (3) compare their leaching products in freshwater and saline water treatments.

## Methods

### Study sites and spatial extent estimates

Deposits of iron and steel slags in the coastal zone were identified by screening a UK-wide database of slag disposal locations (collated from a range of map-based and industrial archaeological sources: see Riley et al. 2020, 2022) to identify all those falling within 100m of the mean high water mark using ArcGIS 10.8. The volume of the heaps was calculated as per Riley et al. (2020) and age range of deposition determined from analysis of historical maps. The coastal frontage (i.e. length of heap falling within Mean High Water mark) was estimated using the ArcGIS intersect tool to give a length in kilometres for each heap. All sites were also screened against a range of natural and cultural conservation designations (Local Nature Reserves, Sites of Special Scientific Interest, National Nature Reserves, Special Protected Areas, Special Areas of Conservation and Ramsar wetland sites) to evaluate potential management considerations at disposal sites. These were screened following the methods of Crane et al. (2017) and Riley et al. (2020) for both direct intersection of slag heaps with conservation sites and a proximity analysis. Sample sites were visited between February 2020 and August 2022 to undertake (a) a walkover of site condition, to document disposal settings, evidence of erosion and potential pathways of material transport from heaps, and (b) to gain representative samples for mineralogical analyses and leaching tests. The stability survey was executed from a walkover of the base of the heaps on the shoreline and from the top of slag heaps. Slag material was often present in the form of unconsolidated particles, typically ranging from approximately 50–100 mm in their longest axis. For each site, a slag sample was collected from the surface every 10 m over a 50-m transect to reduce the influence of any spatial heterogeneity within composition across a site, and then aggregated to represent one bulk sample. This procedure was repeated three times, resulting in three replicate samples of material for each site.

### Slag composition

#### Major and trace elemental composition

The major elemental composition of slag samples was determined using a quantitative fused-bead X-ray fluorescence (XRF) method. Slag samples were air-dried in ambient conditions until thoroughly dry, after which a fly press and disc mill were used to crush and pulverise samples to obtain a homogeneous powder. Loss on ignition (LOI) analysis was performed at 1025 °C, after which a subsample of slag was mixed with a flux (66 % Li_2_B_4_O_7_, 34 % LiBO_2_) at a sample to flux ratio of 1:10 and melted to produce a fused glass bead. For trace elemental analysis, a separate subsample of each slag was mixed with a CEROX binder at a ratio of 4:1, and then pressed at 10 tonnes in a 32 mm die to produce a pellet. The bead and pellet were then analysed using a Rigaku ZSX Primus II XRF spectrometer at the University of Leeds to determine elemental composition. During analysis, certified reference materials (BIR-1; STSD-4; MRG-1; STM-1) were repeatedly measured; for these standards, major and trace elemental analysis was typically within 3% and 7% of standard values, respectively.

#### Mineralogy

The determination of major mineral phases within six different slag samples (selected to represent a regional coverage of GB) was achieved using X-ray diffraction (XRD). This was performed by placing powdered slags into Al holders, then analysing with a Cu Kα radiation source operating at 35 kV and 40 mA. Samples were scanned from 2 to 86 °2 θ, at a step size of 0.02 °2 θ, with a counting time of 1 s per step, using a Bruker D8 diffractometer. Diffraction patterns were analysed using the EVA software and the ICDD PDF2 database. Further to this, scanning electron microscopy (SEM) imaging was performed using a Tescan VEGA3 XM equipped with an X-max 150 SDD energy dispersive x-ray spectrometer (EDS). A beam energy of 15 keV at a distance of 15 mm was used to visually map elemental composition and mineral phases at a spatial resolution of 2 μm (as per Pullin et al. 2019).

#### X-ray absorption spectroscopy (XAS)

Selected samples of the predominant blast furnace slags were chosen for detailed analysis of Cr and V valence to complement mineralogical characterisation given these elements are often the principal concern for leaching from slag deposits (Chaurand et al. 2007; Hobson et al. 2017). Prior to analysis, 1 g samples of slag from sites Barrow Haven (BHA) and Ulverston slag bank (ULV) were set within epoxy resin (Epofix) to produce a 30-mm diameter block, backed with araldite resin to achieve a final block thickness of 15 mm. The blocks were polished to a 1 μm finish, with final polishing achieved using a diamond-based solution. These samples were selected as they are representative of the dominant mineralogies and elemental composition present in coastal slag deposits (see Results section). XAS was performed using the I18 Beamline at the Diamond Light Source synchrotron, Oxfordshire, UK (Mosslemans et al. 2009). Micro-XRF (μXRF) elemental mapping allowed for visualisation of elemental distribution within each sample, and informed the locations for subsequent analysis. The oxidation state of elements within slag samples was measured using micro-X-ray absorption near edge structure (μXANES) spectroscopy, with spectra collected at the Cr and V K-edges (5989 and 5465 eV, respectively). For the ULV sample, μXANES data were collected from within ‘hotspots’ of high Cr and V concentration in the block (Supporting Information Figure 1), whereas spectra were collected from random spots on the BHA block given its uniform composition in μXRF maps. XAS spectra were collected from Cr (FeCrO_4_, Cr_2_O_3_) and V (V_2_O_3_, VOSO_4_) standards to compare with sample data. Standards were prepared as pressed pellets diluted with cellulose and XAS spectra were collected in transmission mode on the I18 Beamline.

### Leaching tests

**Fig. 1:**
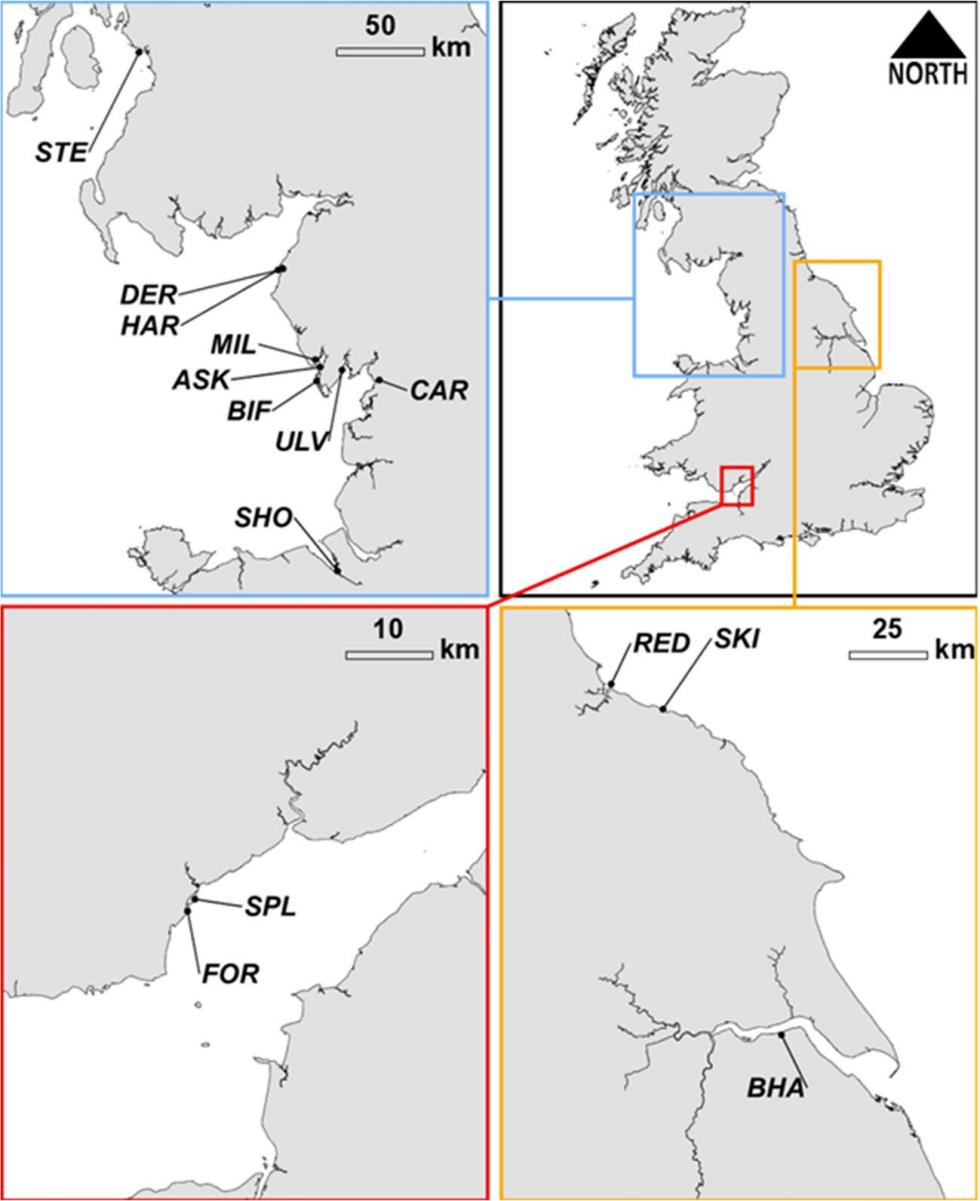
Locations of coastal slag deposits identified in this study (detailed information in Table 1)

**Fig. 2:**
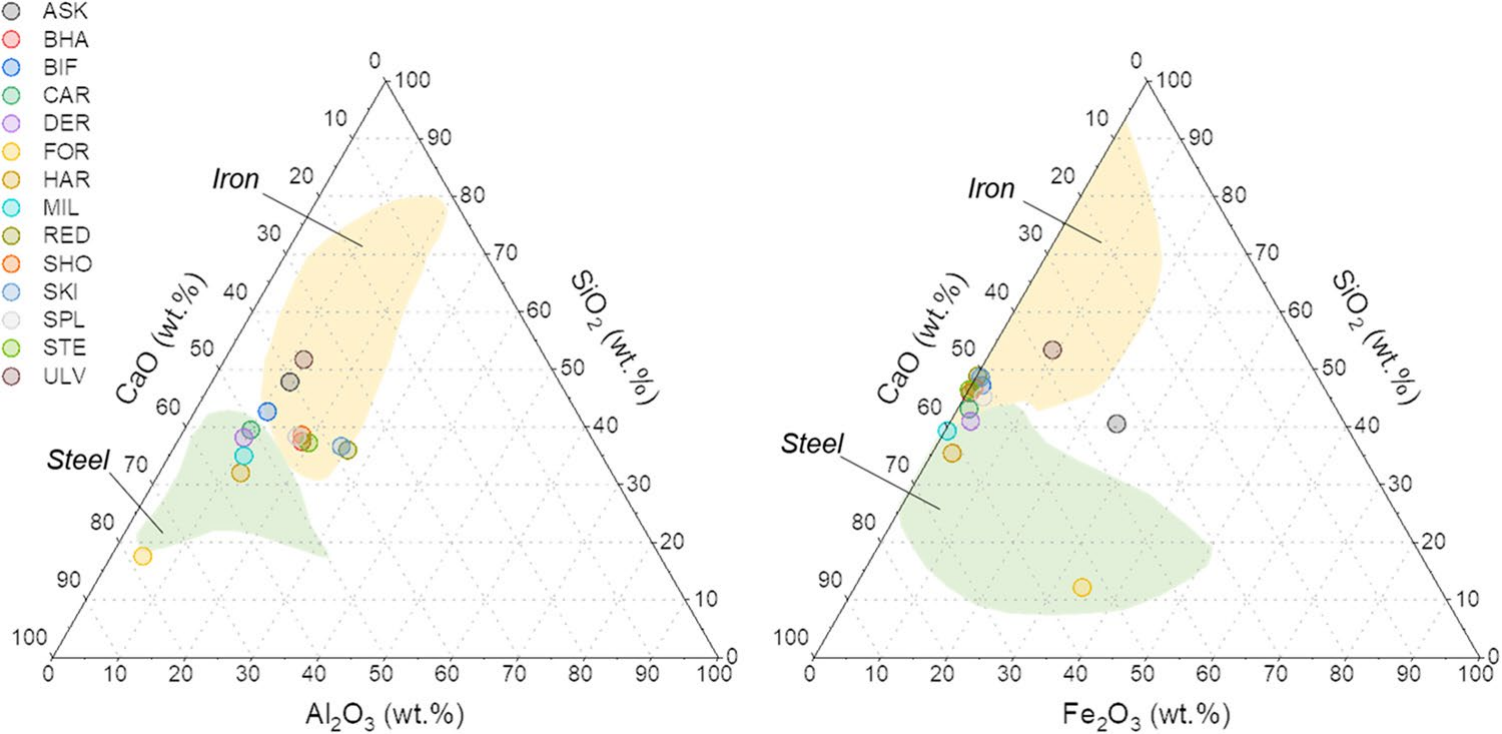
Ternary diagrams for classification of slags by site based on; Al_2_O_3_, SiO_2_ and CaO end members (left), and Fe_2_O_3_, SiO_2_ and CaO end members (right). Yellow and green shaded areas represent typical plotting regions for iron (blast furnace) and steel (basic oxygen furnace) slags, respectively (after Piatak et al. 2015)

Slag samples from five locations (BHA, DER, RED SHO, STE; Fig. 1) were subject to leaching tests based on the British Standard method BS EN 12457-2 (British Standards Institution 2002), a standard compliance test for leaching of granular waste materials which has been used in other studies of waste leachability (Brand and Spencer 2020). These sites were selected as their composition was typical of slags collected nationally, as seen by their relative grouping within ternary plots (Fig. 2). Samples from Forty Acre site (FOR) were not subject to leaching tests given that BOF slags were not typical of coastal slags encountered more widely around the UK, and the relatively higher understanding of BOF leaching in current literature (e.g. Hobson et al. 2017). In triplicate, 90 g (± 5 g) of crushed slag (< 4mm grain size) from each site was contacted with a deionised water (DI) leachant (pH 7.41) at a liquid to solid ratio of 10 L/kg slag. Immediately after contact (*t = 0*), a 10 mL sample was collected and filtered (0.45 μm) for analysis, and measurements of pH, oxidation reduction potential (ORP) and conductivity were taken using a Myron L Ultrameter. The mixture was then placed on an end-over-end rotator for 24 h (*t = 24*) at 10 RPM. This method was repeated using a seawater leachant. Seawater was collected from the North Sea at South Landing, Flamborough, North Yorkshire (54°06′14″N, 000°07′09″W) to represent typical UK coastal waters away from large point pollution sources or areas of industrial activity, and were filtered at 0.45 μm to remove sand and marine debris. For each leachant type, a blank sample containing no crushed slag was also processed in the same manner. After 24 h, the mixtures were removed from the rotator, allowed to settle, and were sampled and measured for pH, ORP and conductivity using a Myron L Ultrameter. All leachate samples were acidified using minimal addition of 70% HNO_3_ (99.999% trace metals basis), refrigerated and subsequently analysed by an external commercial laboratory (Socotec) using ICP-OES and ICP-MS analysis for deionised water and seawater samples, respectively. Statistical differences between average concentrations of key parameters in deionised and seawater treatments were tested using Mann-Whitney (given data did not conform to a normal distribution) using R programming language.

## Results

### Extent

There was in excess of 49 million m^3^ of iron and steel slag deposited within coastal sites identified in this study. Figure 1 and Table 1 highlight the distribution of these deposits, with the majority of this total (~80%) situated within the north west of England on the Lancashire and Cumbria coast. The 14 identified slag deposits also comprise around 75 km of coastline in either deliberate coastal defence structures (around 53 km: e.g. south bank of the Humber estuary in eastern England) and deposits that form incidental coastal frontage and defences (around 22 km). The majority of the deposits dated from the early to mid-20th Century. The slag deposits cover a range of depositional settings from clifftop locations (e.g. Skinningrove (SKI)), artificial peninsulas of slags directly dumped in estuarine settings (e.g. Redcar (RED), BHA, Stevenston Pier (STE), Millom Pier (MIL) and Askam Pier (ASK)), and large deposits creating anthropogenic cliffs on high-energy coastlines (e.g. Derwent Howe (DER), Harrington (HAR)). Erosional features were apparent at a number of sites, ranging from tension cracks in slag banks where collapse is induced by undermining of tidal sediments beneath (e.g. CAR - see Supplementary Information Figure 4), to extensive weathering features including wave-cut notches, caves and arches at high-energy coastlines (e.g. DER, HAR) akin to features more commonly apparent in natural chalk cliffs (see Supplementary Information Figure 4). All but one site (STE) were directly co-located within or immediately adjacent to formal environmental conservation designations.

**Table 1:** Site details, volume and field observations for sampled coastal slag deposits

Site	Site code	Volume (m^3^) (from Riley et al. 2020)	Coastal frontage length (km)	Approximate age of deposit	Forms part of coastal defences? (Y/N - Intentional/incidental)	Field observations (e.g. habitat creation, erosion)
Askam Pier	ASK	1,414,827	1.4	1890s–1910s	Y - incidental	Artificial peninsula made of directly tipped slag. Part of Duddon Bay Ramsar wetland site, Site of Special Scientific Interest (SSSI), Special Area of Conservation (SAC), and Special Protection Area (SPA).
Barrow Haven	BHA	NA	0.3*	1910s–1950s	Y - intentional	*Artificial peninsula of BF slag sampled; forms part of 49 km tidal flood defence barrier (bitumen coated). Within the Humber Estuary Ramsar site, SSSI, SAC and SPA.
Barrow-in-Furness	BIF	8,255,077	1.5	1890s–1970s	Y - incidental	Partly reworked on the landward side for aggregate. Potential encapsulation of other industrial wastes. Part of North Walney National Nature Reserve (NNR) and Duddon Estuary Ramsar site, SSSI, SAC and SPA.
Carnforth Slag Bank	CAR	962,699	2.8	1860s–1890s	Y - incidental	Offers erosional protection to landward saltmarsh habitat and MSW landfill; some evidence of undermining due to erosion of tidal flats; part of Morecambe Bay SAC, SPA and Ramsar site.
Derwent Howe	DER	26,729,599*	4.1	1920s–1960s	Y - incidental	Tall cliff deposits (>8m high). Evidence of extensive erosional features, with hardpans over deposits below mean high water. Situated within the Solway Firth SPA.
Harrington Slag Bank	HAR					Extensive erosional features and undercutting. Within the Solway Firth SPA.
Forty Acre, Cardiff	FOR	1,263,020	1.5	1950s–1980s	Y - incidental	Directly tipped slag in estuarine setting. Used in part as land reclamation. More recent MSW and C&D landfill deposited on slag. Evidence of erosion and management (rock armour) in places. Within the Severn Estuary Ramsar site, SSSI, SAC and SPA.
Millom Pier	MIL	1,731,091	2.1	1960s–1980s	Y - incidental	Forms part of Duddon Estuary Ramsar wetland, SSSI, SAC, SPA and ground nesting bird habitat. Also forms Millom Ironworks Local Nature Reserve (LNR).
Redcar Breakwater	RED	1,347,926	4.4	1870s–1910s	Y - intentional	Part of Teesmouth and Cleveland Coast SPA, SAC, Ramsar site and SSSI.
Shotton Rail Sidings	SHO	372,015	2.2	1960s–1980s	N	Forms part of Shotton Lagoons and Reedbed SSSI.
Splott, Cardiff	SPL	5,504,455	2.0	1950s–1980s	Y- incidental	Extensive direct tipping of molten slag in estuary and reworked material. Significant undercutting and erosion in places. Within the Severn Estuary Ramsar site, SSSI, SAC and SPA.
Skinningrove Cliffs	SKI	NA	0.2	1920s–1970s	N	Clifftop deposit with some slag used in breakwater.
Stevenston Pier	STE	568,245	1.5	1900s–1940s	Y - incidental	~5 m high promontory created through slag tipping onto sandy beach.
Ulverston Slag Bank	ULV	2,801,749	1.7	1900–1950	Y - incidental	Part of Morecambe Bay SAC, SPA and Ramsar site.

### Composition

Compositional XRF data were converted to oxide equivalent concentrations (wt. %) and used to generate Al_2_O_3_-SiO_2_-CaO and Fe_2_O_3_-SiO_2_-CaO ternary plots to determine the likely origin of slags based on major elemental composition (Fig. 2). Using the classifications specified by Piatak et al. (2015), in most cases, samples were more closely aligned with iron-making blast furnace slags. Exceptions to this were the samples from HAR, MIL and DER, which were more similar in composition to steel-making slags. For ASK, whilst plotting within the iron-making slag region in the Al_2_O_3_-SiO_2_-CaO plot, its position in the Fe_2_O_3_-SiO_2_-CaO plot (outside of either region) was the result of very high Fe concentrations within some sub-samples, as shown by the high variability of this measurement in Table 2 and Supporting Information Figure 2. Other major phases present include K, Mn and Ti (typically between 0.5 and 1 wt.%, Table 2). Given that slags are high temperature wastes (~1500 °C) when deposited, any moisture within slag samples is a result of slag hydration and water uptake post-deposition. Moisture content analysis (Table 2) suggested a slight negative relationship between LOI and Mg concentration (known to be preferentially leached during slag weathering) (Gomes and Pinto 2006), whereby slags with higher LOI values tended to have lower Mg concentrations, potentially a sign of weathering within these samples (particularly from HAR, CAR and ASK). Trace element composition showed consistently low concentrations of many elements of potential interest (Table 3). The highest Cr (5988 mg/kg) and V (2888 mg/kg) concentrations were in slags from FOR, though note that as BOF type slags, these were not representative of most coastal slag deposits encountered. Concentrations of As were below detection limits at all but four Cumbrian and Lancastrian sites (ASK, CAR, HAR, ULV), whilst Cu, Pb and Zn showed some variability across sites, but with concentrations typically in the low 10s of mg/kg. Ba and Sr enrichment was apparent at some sites (typically those with high Ca content) reaching values in excess of 9000 and 700 mg/kg, respectively (Table 3).

**Table 2:** Major elemental composition of slag samples (wt.% oxide equivalent) and loss on ignition (%) by site (< LOD = below XRF instrument detection limits)

	SiO_2_	CaO	Al_2_O_3_	MgO	Fe_2_O3	K_2_O	MnO	TiO_2_	Na_2_O	P_2_O_5_	LOI	Total
*SITE*	*wt.%*	*wt.%*	*wt.%*	*wt.%*	*wt.%*	*wt.%*	*wt.%*	*wt.%*	*wt.%*	*wt.%*	*wt.%*	*wt.%*
ASK	30.4 *(± 6.4)*	25.7 *(± 8)*	7.5 *(± 2)*	2.6 *(± 0.3)*	18.8 *(± 12)*	0.6 *(± 0.3)*	0.7 *(± 0.2)*	0.2 *(± 0.1)*	0.3 *(± 0.1)*	*< LOD*	10.8 *(± 3.8)*	97.6 *(± 0.8)*
BHA	33.2 *(± 0.6)*	38.8 *(± 0.4)*	16.6 *(± 0.2)*	3.7 *(± 0)*	0.3 *(± 0.1)*	1.6 *(± 0.1)*	1.2 *(± 0.1)*	0.6 *(± 0)*	0.3 *(± 0)*	0.1 *(± 0)*	0.7 *(± 0.1)*	97.2 *(± 0.2)*
BIF	36 *(± 2)*	39.1 *(± 1.5)*	9.3 *(± 0.4)*	1.9 *(± 0.7)*	1.2 *(± 0.8)*	0.4 *(± 0.1)*	1.2 *(± 0.1)*	0.4 *(± 0.1)*	0.3 *(± 0.2)*	*< LOD*	6.9 *(± 2.5*)	96.7 *(± 0.2)*
CAR	30.8 *(± 2.5)*	39.3 *(± 2)*	7.8 *(± 0.4)*	1.1 *(± 0.1)*	1.2 *(± 0.9)*	0.4 *(± 0.2)*	0.4 *(± 0.2)*	0.4 *(± 0.1)*	0.1 *(± 0)*	*< LOD*	14.5 *(± 4.8)*	95.9 *(± 0.7)*
DER	31 *(± 1.5)*	42.3 *(± 2)*	7.9 *(± 0.4)*	3.4 *(± 0.4)*	2.3 *(± 1)*	0.1 *(± 0)*	0.7 *(± 0.1)*	0.3 *(± 0.1)*	0.4 *(± 0.2)*	*< LOD*	4.1 *(± 2.9)*	92.5 *(± 1)*
FOR	8.3 *(± 0.7)*	36.7 *(± 1.4)*	2.3 *(± 0.4)*	13.0 *(± 2.8)*	23.4 *(± 0.5)*	*< LOD*	5.3 *(± 0.2)*	0.3 *(± 0.0)*	0.1 *(± 0.0)*	*4.1* *(± 0.2)*	0.4 *(± 0.2)*	94.2 *(± 1.1)*
HAR	22.7 *(± 0.6)*	39.4 *(± 2)*	8.7 *(± 0.3)*	1.9 *(± 0.1)*	1.9 *(± 0.6)*	0.1 *(± 0)*	*< LOD*	0.5 *(± 0)*	0.2 *(± 0)*	*< LOD*	19.4 *(± 2.6)*	94.7 *(± 0.5)*
MIL	29 *(± 1.2)*	44.5 *(± 2.4)*	9.3 *(± 0.4)*	3.7 *(± 0.7)*	0.2 *(± 0.1)*	0.3 *(± 0.1)*	0.3 *(± 0.1)*	0.3 *(± 0.1)*	0.1 *(± 0)*	*< LOD*	10.4 *(± 4.2)*	98.3 *(± 1.3)*
RED	30.5 *(± 0.8)*	31.9 *(± 0.5)*	22.3 *(± 0.8)*	9.4 *(± 0.6)*	0.1 *(± 0)*	0.5 *(± 0.2)*	0.4 *(± 0)*	0.5 *(± 0)*	0.7 *(± 0.2)*	*< LOD*	1.7 *(± 0.6)*	97.9 *(± 0.1)*
SHO	31 *(± 0.5)*	34.8 *(± 0.3)*	14.6 *(± 1.1)*	11.4 *(± 0.7)*	0.4 *(± 0.1)*	0.4 *(± 0.1)*	0.7 *(± 0.1)*	0.6 *(± 0)*	0.3 *(± 0)*	*< LOD*	3.1 *(± 1.1)*	97.3 *(± 0.2)*
SKI	31.2 *(± 0.4)*	32.5 *(± 0.5)*	21.3 *(± 0.2)*	7.9 *(± 0.1)*	0.4 *(± 0)*	1.0 *(± 0)*	0.3 *(± 0)*	0.5 *(± 0)*	0.5 *(± 0)*	*< LOD*	1.6 *(± 0.8)*	97.2 *(± 0.3)*
SPL	31.8 *(± 0.9)*	36.7 *(± 0.7)*	14.6 *(± 1.1)*	7.8 *(± 1.4)*	2.0 *(± 0.7)*	0.3 *(± 0.1)*	0.8 *(± 0.3)*	0.3 *(± 0.1)*	0.3 *(± 0.0)*	*< LOD*	4.9 *(± 1.6)*	97.6 *(± 0)*
STE	32.9 *(± 0.2)*	37.8 *(± 0.5)*	17.5 *(± 0.5)*	5.6 *(± 0.5)*	*< LOD*	0.5 *(± 0)*	0.6 *(± 0)*	0.3 *(± 0)*	0.2 *(± 0)*	*< LOD*	2.1 *(± 0.1)*	97.7 *(± 0.1)*
ULV	43.8 *(± 1.5)*	30.8 *(± 3.7)*	10.1 *(± 1.2)*	2 *(± 0.5)*	7.5 *(± 3.7)*	0.8 *(± 0)*	1.1 *(± 0.5)*	0.5 *(± 0.1)*	0.3 *(± 0.1)*	*< LOD*	0.6 *(± 0.5)*	97.7 *(± 0.3)*

**Table 3:** Trace elemental composition (mg/kg) of slag samples by site (< LOD = below XRF instrument detection limits)

	As	Ba	Ce	Cr	Cu	La	Nd	Pb	Rb	Sc	Sr	V	Zn	Zr
*SITE*	*mg/kg*	*mg/kg*	*mg/kg*	*mg/kg*	*mg/kg*	*mg/kg*	*mg/kg*	*mg/kg*	*mg/kg*	*mg/kg*	*mg/kg*	*mg/kg*	*mg/kg*	*mg/kg*
ASK	23.3 *(± 16.6)*	1476 *(± 537.6)*	58.3 *(± 14.4)*	21.3 *(± 8*)	31.3 *(± 11.7)*	36 *(± 8)*	28 *(± 5.9)*	31.3 *(± 13.8)*	17.2 *(± 6.2)*	*< LOD*	220 *(± 28.7)*	33.3 *(± 17)*	52.3 *(± 32.1)*	109.7 *(± 32.7)*
BHA	*< LOD*	321.3 *(± 5.2)*	276.7 *(± 6.1)*	38.7 *(± 2.3)*	8.3 *(± 0.3)*	109.3 *(± 1)*	133 *(± 2.2)*	6.7 *(± 0.7)*	54.7 *(± 3.1)*	16 *(± 0.5)*	730.7 *(± 18.4)*	177.3 *(± 10.8)*	9.5 *(± 3)*	213.3 *(± 9.4)*
BIF	*< LOD*	2328 *(± 344.6)*	71.3 *(± 2.7)*	10.3 *(± 4.4)*	5.8 *(± 1.4)*	51 *(± 2.5)*	36 *(± 1.6)*	4 *(± 1.2)*	15.3 *(± 6.8)*	*< LOD*	355.7 *(± 76.9)*	22.3 *(± 8.5)*	6.8 *(± 2.3)*	93 *(± 3.3)*
CAR	4.3 *(± 1.1)*	747.5 *(± 192)*	69.3 *(± 4.2)*	*< LOD*	5.8 *(± 0.6)*	40.3 *(± 3.4)*	43.5 *(± 4.7)*	4.3 *(± 0.9)*	8.8 *(± 4.5)*	*< LOD*	341 *(± 31)*	*< LOD*	3.6 *(± 1)*	118.5 *(± 2.8)*
DER	*< LOD*	9255 *(± 2589.9)*	56 *(± 4.5)*	*< LOD*	*< LOD*	28 *(± 0.9)*	26.7 *(± 0.3)*	*< LOD*	*< LOD*	*< LOD*	615.7 *(± 74)*	11.7 *(± 3.3)*	*< LOD*	82.3 *(± 7.5)*
FOR	*5.9* *(± 0.1)*	811.4 (± 112.9)	9.3 (± 3)	*5988* *(± 1297)*	*96.9* *(± 4.4)*	13.6 (± 1.1)	3.6 (± 1.3)	*18.9* *(± 0.6)*	*1.8* *(± 0.3)*	*12* *(± 1.3)*	226.3 (± 13.1)	2888 (± 453.7)	*34.4* *(± 4.3)*	36.7 (± 2)
HAR	6 *(± 0.9)*	1051.3 *(± 15.5)*	55.5 *(± 2.9)*	8.3 *(± 1.6)*	19.3 *(± 3.6)*	34 *(± 1.8)*	30 *(± 1.5)*	5.8 *(± 0.2)*	*< LOD*	*< LOD*	437.3 *(± 18.4)*	10.5 *(± 2.8)*	5.4 *(± 0.9)*	157.3 *(± 5.9)*
MIL	*< LOD*	1943.3 *(± 198)*	66.7 *(± 7.3)*	*< LOD*	7 *(± 0.9)*	40.7 *(± 4.4)*	36.7 *(± 3.9)*	5.2 *(± 1.1)*	4.5 *(± 0.8)*	*< LOD*	279.3 *(± 11.3)*	4.7 *(± 0.3)*	4.7 *(± 1.8)*	106.7 *(± 18.7)*
RED	*< LOD*	697.3 *(± 71.3)*	420.7 *(± 39.6)*	33.3 *(± 3.1)*	3.3 *(± 0.7)*	145 *(± 16.9)*	191 *(± 17.6)*	*< LOD*	19.3 *(± 7.5)*	51.7 *(± 5.7)*	563 *(± 37.2)*	67.3 *(± 9.1)*	3.3 *(± 0.7)*	257.7 *(± 10.5)*
SHO	*< LOD*	1431.7 *(± 86.9)*	161 *(± 16.4)*	23.3 *(± 2.2)*	14.3 *(± 1.8)*	72.3 *(± 4.1)*	71.3 *(± 6.2)*	49.0 *(± 9)*	7.7 *(± 1.4)*	22 *(± 6.6)*	499.7 *(± 20.4)*	33.7 *(± 6)*	74.3 *(± 7.5)*	196.3 *(± 17.3)*
SKI	*< LOD*	452 *(± 32.7)*	337 *(± 11)*	32 *(± 2.9)*	15.3 *(± 6.2)*	113 *(± 4.9)*	148.3 *(± 4.6)*	5.8 *(± 1.4)*	29 *(± 2.2)*	39.7 *(± 2)*	338.7 *(± 11.2)*	84.7 *(± 10.1)*	30 *(± 10.3)*	234 *(± 7)*
SPL	*5.2* *(± 0.6)*	1339 (± 214.9)	142.6 (± 19.3)	79.3 (± 12.6)	44.5 (± 2.6)	67.8 (± 5.1)	64.9 (± 10.8)	26.6 (± 2.3)	9.0 (± 2.0)	41.8 (± 6.5)	757.7 (± 124.5)	39.3 (± 16.3)	81 (± 26.6)	176.8 (± 30.5)
STE	*< LOD*	800.3 *(± 58.5)*	182.3 *(± 25.8)*	*< LOD*	17.5 *(± 7)*	96.3 *(± 11.8)*	86.7 *(± 10.1)*	*< LOD*	10.7 *(± 1.4)*	6.8 *(± 2.6)*	561.3 *(± 53.6)*	7.3 *(± 1.9)*	5.2 *(± 1.1)*	121.3 *(± 10.7)*
ULV	5 *(± 1.1)*	1009.8 *(± 397.2)*	53.3 *(± 11.3)*	121.5 *(± 60.6)*	22.3 *(± 5)*	34.3 *(± 9.7)*	24.3 *(± 6.1)*	7.8 *(± 2.8)*	22.5 *(± 1.3)*	3.4 *(± 0.8)*	219 *(± 17.2)*	139.5 *(± 41.3)*	15 *(± 6.3)*	104.5 *(± 9.3)*

### Mineralogy

**Fig. 3:**
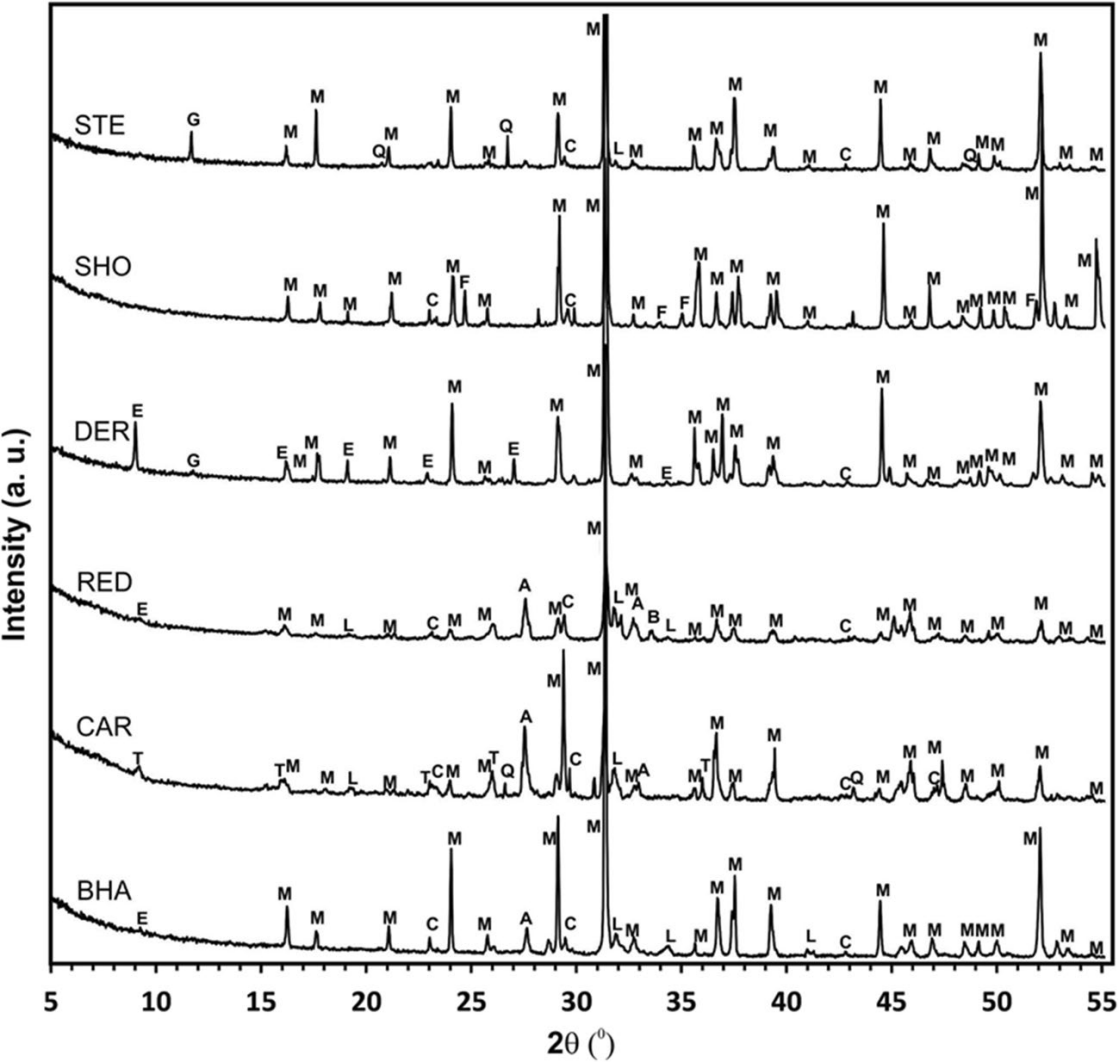
XRD spectra of selected slag samples. A, Anorthite (CaAl_2_Si_2_O_8_); B, brownmillerite (Ca_2_FeAlO_5_); C, calcite (CaCO_3_); E, ettringite (Ca_6_Al_2_OH_12_(SO_4_)_3_.26H_2_O); G, gypsum (CaSO_4_.2H_2_O); L, larnite (β-Ca_2_SiO_4_); M, melilite group (gehlenite – åkermanite solid solution; Ca_2_Al[AlSiO_7_] - Ca_2_Mg[Si_2_O_7_]); Q, quartz (SiO_2_); T, thaumasite (Ca_3_Si(OH)_6_(CO_3_)(SO_4_)·12H_2_O)

**Fig. 4:**
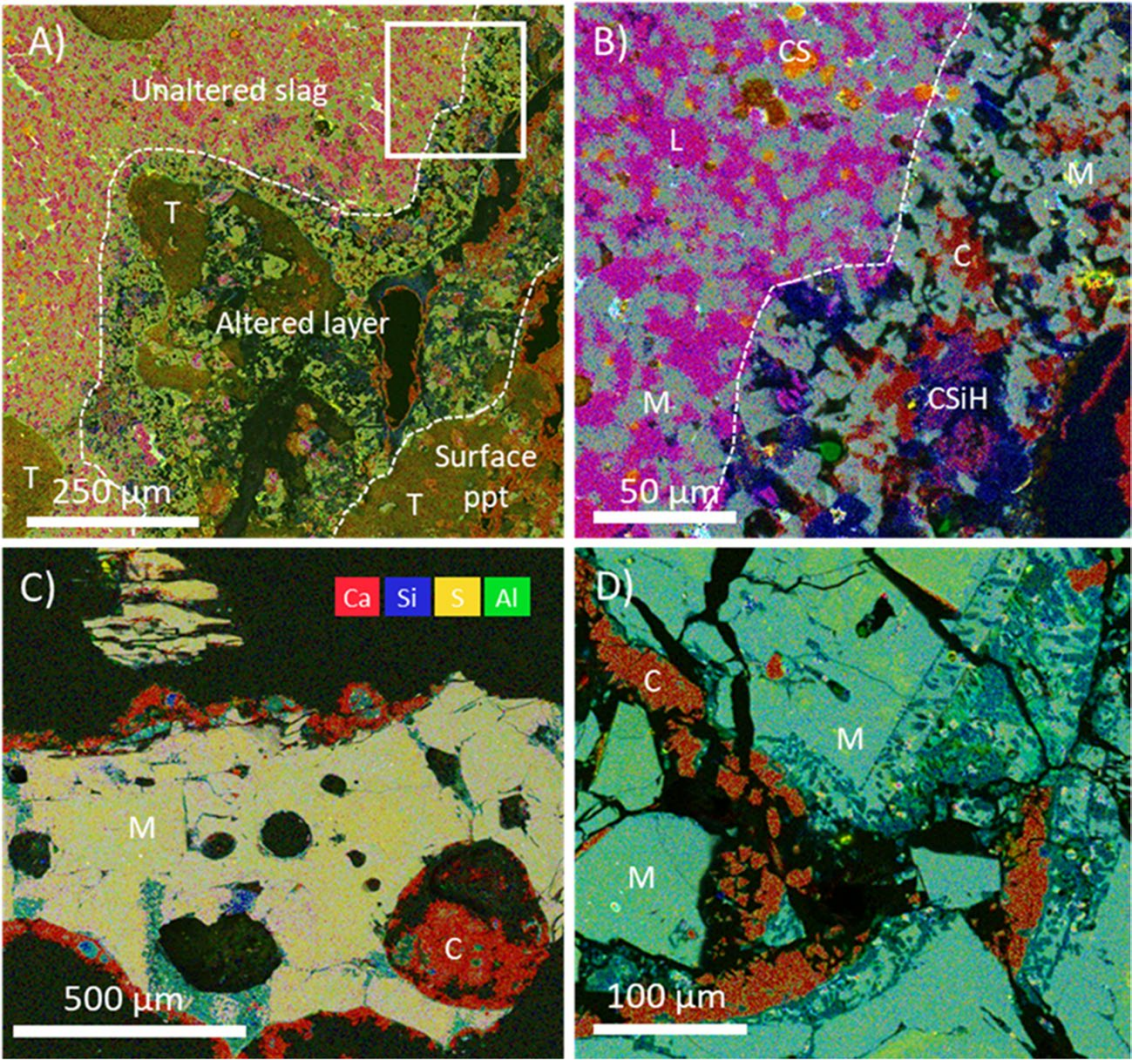
False colour SEM-EDS overlay maps (back-scattered electron images; Ca, Si, S, Al) of slag particles within; **A** DER slag; **B** higher-resolution image of DER (area marked with white box in **A**); **C** and **D** particles within RED slag. Key to mineral phases: L, larnite; CS, calcium sulphide; CSiH, calcium silicate hydrate; M, unaltered melilite; C, calcite, T = thaumasite

XRD patterns (Fig. 3) from the six slag samples analysed were all similar, with multiple overlapping peaks. The absence of broad peaks in the XRD spectra indicated no evidence of significant amorphous or glass phases in any sample. The major mineral phases identified by XRD belonged to the melilite group of Ca silicates of Al and Mg; principally gehlenite, which was identified in all samples. XRD spectra for the melilite group phases overlap considerably, and therefore have not been separated in Fig. 3. Minor phases within the samples have been identified using their primary and secondary peaks, and included phases previously reported for iron-making slags such as larnite, brownmillerite, quartz and anorthite. Secondary phases that form from silicate hydration and carbonation such as calcite, gypsum, ettringite and thaumasite, were also found in some samples. Detailed SEM-EDS overlay maps were generated for samples from DER and RED slags (Fig. 4). DER slag was found to contain several highly reactive phases (e.g. larnite and Ca sulphide), where weathering has produced an altered surface layer containing Ca silicate hydrate, unaltered melilite and precipitated mineral phases such as calcite and thaumasite. Samples from RED were primarily composed of melilite phases, and showed no discernable altered layer. However, surface calcite precipitation was observed on particles, which also formed in intra-particle cracks and fissures.

### XANES

**Fig. 5:**
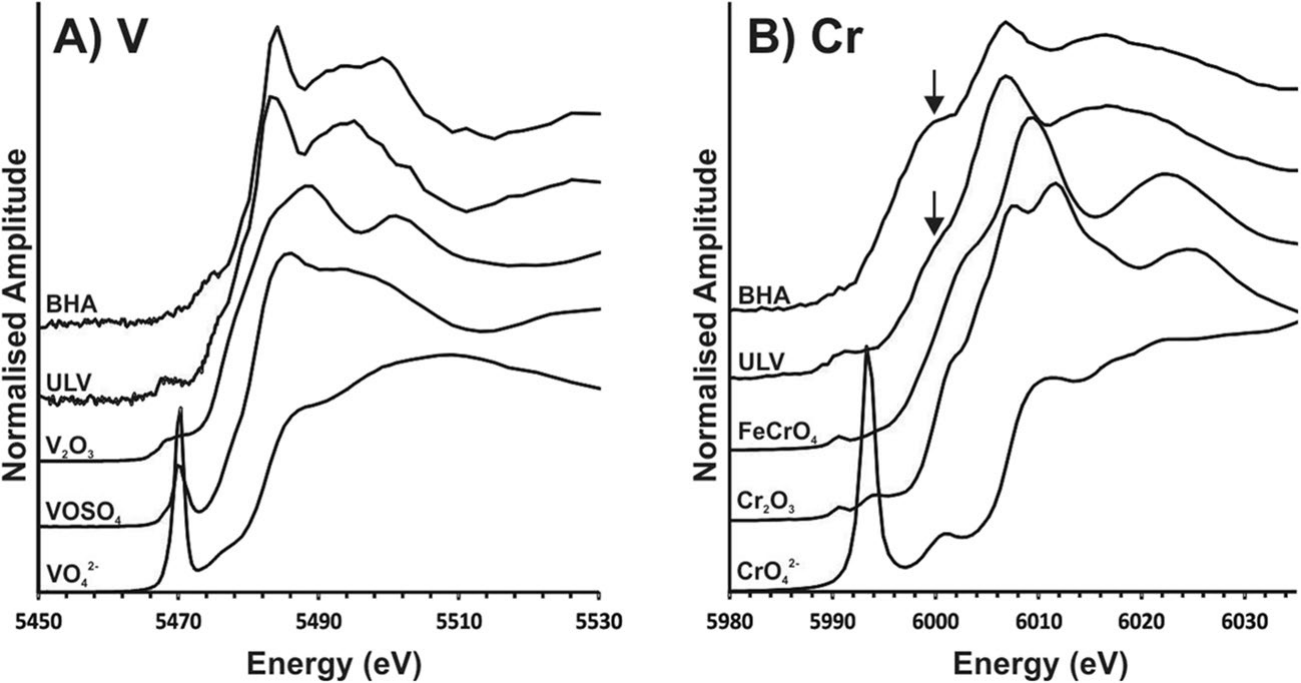
Averaged XANES spectra of V and Cr edges in aggregated samples from Barrow Haven (BHA) and Ulverston (ULV). Arrows on the Cr spectra show the position of the primary absorption peak present in Cr(III)- sulphide XANES spectra

Of particular concern in slags are V and Cr, which are both able to form toxic and environmentally-mobile oxyanions (i.e. V(V) and Cr(VI)) under high pH settings (Hobson et al. 2018). As such, their chemical oxidation state was determined using XANES analysis (Fig. 5) for samples from Barrow Haven (BHA) and ULV given that these sites were largely representative of slags from other areas, but exhibited relatively high V and Cr concentrations at ranges suitable for XAS synchrotron analysis (Table 3). For both sites, the averaged V spectra were similar, as was also true for Cr, despite differences in sample structures. Both V and Cr spectra lacked evidence of pre-edge peaks, which are indicative of higher valence forms, and so both are more likely to be dominated by their trivalent forms (i.e. V(III) and Cr(III)). This is further implied for Cr by the features marked by arrows at ~6000 eV, which are at the correct energy position to be consistent with Cr(III)-sulphide (Hibble et al. 1996), although the sample spectra were not well-matched to the standards analysed, and so, precise speciation is uncertain.

### Leaching tests

**Fig. 6:**
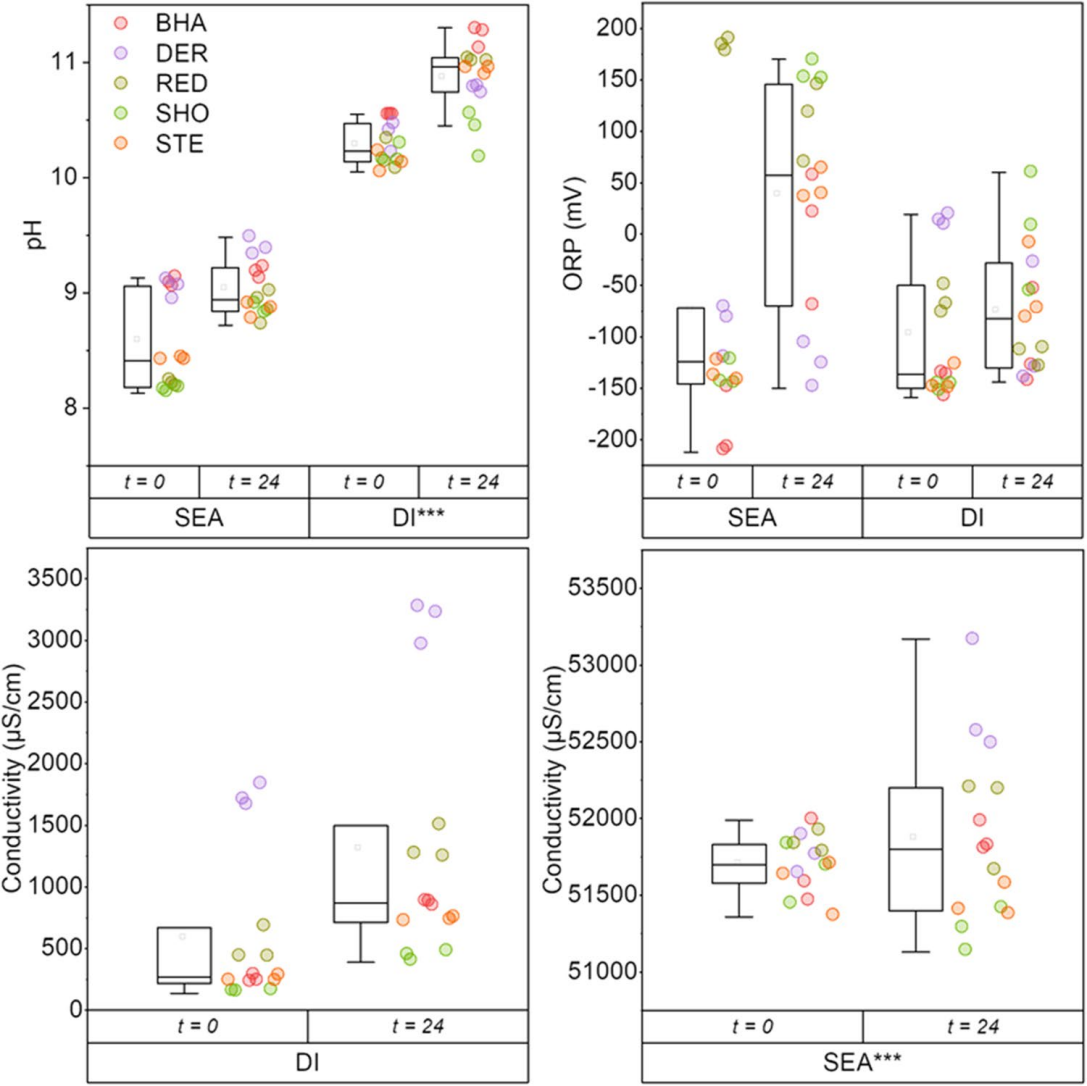
Aggregated pH, ORP (mV) and electrical conductivity (μS/cm) measurements of leachates immediately after contact with slag (*t=*0), and after 24 h (*t=24*). Statistically significant differences (SEA vs DI) are marked by asterisk on higher result (Mann-Whitney test, ***p <0.001)

Physicochemical parameters (pH, ORP, electrical conductivity) were measured immediately after contact with crushed slags, and again after 24 h contact time. For both the deionised (DI) and seawater leachants, an immediate increase in pH was observed on contact with slag, which increased over the 24-h period (Fig. 6). This behaviour was more pronounced in the deionised water leachant, increasing to pH 11 after 24 h, whereas the increase in seawater was less extreme, reaching pH 9. For the seawater leachant, the prolonged contact with most slag samples led to more-oxidising conditions, as seen by the generally higher measured ORP values after 24 h in Fig. 6, though there was large variation in final ORP measurements with some leachates being in reduced conditions and others in oxidising conditions (range −210 to 175 mV), suggesting some variation in leaching behaviour of slags from different locations. Whilst there were differences in slag leaching behaviour from different sites when using a deionised water leachant, the overall range of ORP values was similar after 24 h to the measured conditions at 0 h (range −150 to 50 mV). The electrical conductivity of leachates from deionised waters showed modest increases directly upon contact with the crushed slag, and were notably higher after 24 h, though there was again a lot of variation between sites (range: 100–3300 μS/cm). For seawater leachates, whilst conductivity measurements were substantially higher given the high salinity of seawaters (mean blank EC: 51,500 μS/cm), increases in electrical conductivity as a result of slag leaching were relatively modest, with mean measurements increasing by ~500 μS/cm.

**Fig. 7:**
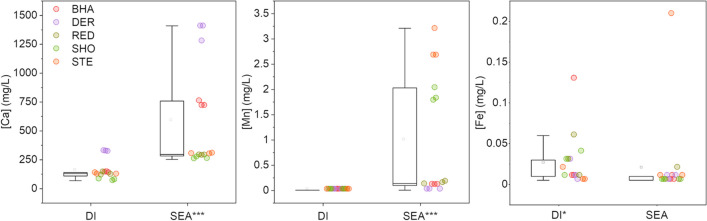
Leached concentrations (mg/L) of Ca, Mn and Fe in deionised water (DI) and seawater (SEA) matrices. Statistically significant differences marked by asterisk on higher result (Mann-Whitney, ***p <0.001, *p <0.05)

Fig. 7 presents the concentrations of major elements in solutions after 24 h contact time. The leaching of Ca from slags is well-documented, and is intrinsic to the weathering process (Mayes et al. 2018). The differential leaching of Ca from slags in deionised and seawater matrices is apparent in Fig. 7, where substantially higher release (mean ~600 mg/L) was observed in seawater conditions, albeit with more variability than in deionised water (mean ~125 mg/L). Release of Mn from slags was much lower than for Ca, with concentrations up to 3.25 mg/L Mn in seawater conditions, but negligible release using the deionised water leachant. Favourable leaching of Ca and Mn was observed in saline waters. However, leaching of Fe was slightly inhibited when using seawater, although the leached concentrations were very low for both matrices (generally < 0.05 mg/L Fe).

**Fig. 8:**
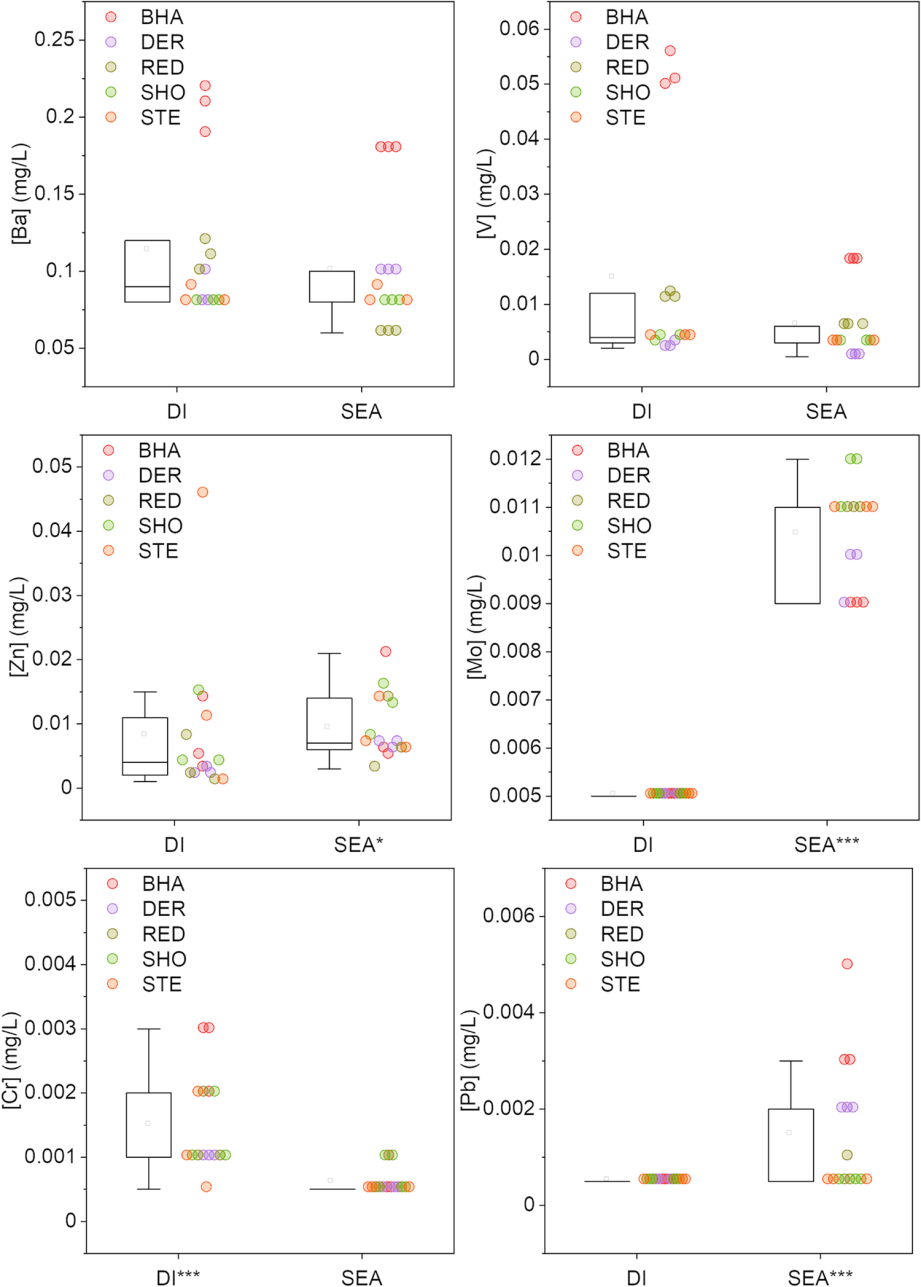
Leached concentrations (mg/L) of selected trace elements in deionised water (DI) and seawater (SEA) matrices. Statistically significant differences marked by asterisk on the significantly higher result (Mann-Whitney, ***p <0.001, *p <0.05)

It is known that during weathering processes, iron and steelmaking slags have potential to release a suite of potentially toxic trace elements (Mayes et al. 2008). When deionised water was used as a leachant, low concentrations of such elements were detected in leachates, specifically Al (range: 0.05–4.1 mg/L), Ba (range: 0.075–0.22 mg/L), V (0.001–0.055 mg/L) and Cr (0.001–0.0025 mg/L; Fig. 8). The release of these elements follows a significant positive correlation with leachant end pH (Supporting Information Figure 3). Neither Pb nor Mo were detected in deionised leachates after 24 h (Fig. 8). Different leaching behaviour was observed in the seawater matrix, where concentrations of Ba, V and Cr were typically lower. A larger, albeit still low, release of Mo was observed (mean, 0.0015 mg/L) in seawater, as was the case for Zn also (mean, 0.0075 mg/L). Data for As, Cd, Hg and Ni were all below detection limits, suggesting little leaching of these contaminants.

## Discussion

### Extent and nature of deposits

The GIS screening identified in excess of 49 million m^3^ of iron and steel slags in the coastal zone of the UK, with incidental disposal sites covering a coastline length of over 25 km (Table 1). This is likely to be a conservative lower estimate given the widespread dumping of slag at sea in near-shore environments around many iron and steel production areas of the UK (Lee 1974; Hamilton 1999) and the poorly documented, but widespread, use of slags in coastal defences at other sites not identified by the screening here. The majority of the coastal slag deposits are concentrated in north west England on the Cumbrian coast. This area has been previously estimated to contain ~60% of all national iron and steelmaking slag deposits (Riley et al. 2022), due to an agglomeration of iron works developed to exploit hematite deposits in the region between the mid-19th Century and the 1980s (Juckes 2002). Significant deposits are also present in other locations of traditional iron and steel production in Teesside, north Lincolnshire and South Wales (Riley et al. 2020; Chukwuma et al. 2021). Where deposits form incidental coastal defences, there appear to be tangible benefits in erosion control in some settings, particularly where slags have lithified through weathering to form cementitious features (MacDonald et al. 2023). For example, at CAR (Lancashire), the extensive BF slag bank protects a municipal waste landfill and area of saltmarsh in a largely soft-sediment coastline. At ASK (Cumbria), the BF slag deposit has formed an artificial peninsula directly in the estuary, which directs the main Duddon Channel away from the shoreline and minimises erosion risk to a number of coastal properties (Cumbria County Council 2018). In high-energy settings, there is extensive evidence of both erosional features in slag deposits (see Supporting Information Figure 4) and of physical transport of slags from their depositional location on land into the intertidal zone (Hamilton 1999).

Many of the sites fall within a range of statutory conservation designations ranging from local significance (e.g. Local Nature Reserve at MIL) to international importance (e.g. Ramsar wetland sites, Table 1), with slag deposits being noted of importance for calcareous grassland plant species and nesting provision in some of the formal designations (Riley et al. 2020). Given these designations, interest in reworking some of the deposits as was noted at some locations—particularly the blast furnace (BF) slags which are all reused as aggregate/cement substitute from modern iron and steel works (Gomes et al. 2016)—needs balancing with in-situ conservation value at some of these sites (Mayes et al. 2022).

### Composition

The composition of the slags shows that the majority of the coastal deposits are BF slags, given the lower Fe content and Ca content than BOF slag (Fig. 2; Proctor et al. 2000; Piatak et al. 2019). Some of the Cumbrian deposits in close proximity to steelworks operating up to the mid-20th Century (DER, HAR, MIL: Riley et al. 2020) are more consistent with BOF composition. The mineralogy of the majority of samples revealed phases within all samples are consistent with being air-cooled iron slags that have been partially weathered (Fig. 3; Hobson et al. 2017; Pullin et al. 2019). The presence of secondary phases such as gypsum, ettringite and thaumasite are all indicative of weathering (Pullin et al. 2019). These were most pronounced in samples from high-energy coastlines, such as DER on the Cumbrian coast, where extensive hardpans were apparent over slag deposits below mean high water (Table 1, Supporting Information Figure 4). The preservation of larnite in samples is notable as it readily hydrates to form Ca-Si-hydrate phases and produces soluble alkalinity which is reflected in some of the higher final pH values in the leaching test data (Figs. 7 and 8; Hobson et al. 2017). Melilite phases such as gehlenite hydrate much more slowly and are considered relatively stable (Khudhur et al. 2024; Stewart et al. 2018).

The presence of a range of minor elements in the slags, such as Ba, Cr, Mn, Pb, V and Zn, is consistent with the reported ranges of BF and BOF slag, generally towards the lower end of quoted values (Proctor et al. 2000; Piatak et al. 2019). These are similar across sites, although elevated alkaline earths (Ba and Sr) correspond with very high Ca concentrations at some Cumbrian sites. The ULV (Cumbria) samples show particular enrichment of Cr and V, whilst the BHA (North Lincolnshire) site shows enriched V, typical of slags derived from Jurassic ironstones (Hobson et al. 2017). Cr and V have received particular attention in iron and steel slags given their potential leachability in forms that can be hazardous to the environment (e.g. Frank et al. 1996) and as such were a focus for additional investigation here using XAS approaches in high concentration samples from BHA and ULV. Cr and V are present in the two analysed slag samples in lower valence (3+) forms that would need to be both weathered from host phases and oxidised to more mobile V(V) and Cr(VI) species to become soluble in seawater. The limited solubility of these elements is evidenced by the low Cr and V concentrations observed in the leaching tests, particularly in seawater scenarios where pH remains buffered below 9.5 (Fig. 6).

### Leaching tests

Leaching with seawater typically moderated trace element release for the majority of elements of potential environmental concern in the slags such as Ba, Cr, Pb, V and Zn. This is likely driven by pH of the leaching tests under the British Standard method BS EN 12457-2 (British Standards Institution 2002), where deionised water treatments attained pH significantly higher (10.1–11.3) than seawater treatments (8.2–9.0) given the buffering capacity of the bicarbonate in seawater (Fig. 6). The pH values documented for deionised treatments are consistent with the weathering of alkalinity-generating larnite phases and give a pH range similar to that documented at slag disposal sites in freshwater settings (e.g. Mayes et al. 2008; Roadcap et al. 2006). The significant positive relationship between leachant end pH and trace element concentration for Al, Ba, Cr and V is typical of solubility-controlled trace element release from alkaline residues across the pH range observed between 8.7–11.3 (Cornelis et al. 2008; Tompkins et al. 2022; Supporting Information Figure 3). This lower trace element release in saline conditions is in contrast to analogous published accounts of slag leaching with seawater which have suggested elevated leaching of trace metals including As, Bi, Cd, Cu, Pb and Zn from smelter slags with increased salinity, albeit pH in the tests was static (Shanmuganathan et al. 2012; Schmukat et al. 2012). Recent studies have also highlighted the value of V as a tracer for leachate from BOF slag in marine systems (Foekema et al. 2021). Whilst there was modest V release in the seawater treatments here, it was at levels typically lower than deionised water treatments, and at concentrations that should not pose significant risks (Foekema et al. 2021). Such V behaviour was consistent with the XANES analysis showing the V in its least mobile, trivalent form (Fig. 5). The only exceptions to this pattern of lower metal(loid) release in seawater treatments observed in this study are for Mo and Mn. Modest enrichment of Mo in the seawater treatments reported here was apparent at levels well below documented aquatic life standards (e.g. 73 μg/L: Fletcher et al. 1997). This leaching of Mo may be related to the excess Ca release under seawater treatments, given Ca-phases (e.g. powellite) are well-documented to control molybdate release from alkaline residues (Cornelis et al. 2008). However, the preferential release of Mn from slags in saltwater treatment conditions is of note. Mn is considered an emerging marine contaminant (Summer et al. 2019), and the concentrations apparent in seawater leaching tests (range: 0.001–3.20 mg/L) fall within the range of documented No Observed Effect Concentrations for a range of marine cnidarians and diatoms (Summer et al. 2019). Although comparing concentrations from leaching tests (using crushed slag material) with real world leaching products must be done with caution, particularly in coastal settings with very high dilution capacities, the monitoring of Mn around coastal BF disposal sites would be of potential value. The preferential leaching of Mn from BOF steel slags in high ionic strength waters has previously been documented by Han et al. (2019) and related to the elevated solubility of Fe and Mn (oxy)hydroxides at high chloride concentrations and the subsequent stability of aqueous Mn-chloride complexes in seawater (Han et al. 2019; Hydes 1980). Whilst statistically significant, the difference in Fe release between deionised water and seawater treatments was much lower than documented elsewhere for BOF slag (Han et al. 2019). This may reflect the much lower initial concentrations of Fe in the predominant BF slag assessed here (typically <5% FeO) compared to previous studies on BOF (~24% FeO: Han et al. 2019).

As such, the leaching data presented here for what are predominantly BF slags indicate similar findings to studies on BOF slag leaching which suggest minimal environmental risk of leaching products in coastal settings (e.g. Foekema et al. 2021), and certainly a lower risk than at inland disposal areas, where hyperalkaline metal-rich leachates have been documented (Roadcap et al. 2006; Mayes et al. 2008). Furthermore, the leaching protocol followed here is likely to represent worst-case scenarios, due to the crushed, fine-grained nature of the slag samples. The presence of secondary mineral phases in weathered samples, particularly the formation of surface carbonate and Ca silicate hydrate phases in some slags (Fig. 4), are likely to further limit trace element release under environmental conditions due to surface armouring, as observed elsewhere (Hobson et al. 2017; Pullin et al. 2019).

## Conclusions

This study has identified in excess of 49 million m^3^ of iron and steelmaking by-products in the coastal zone of the UK across fourteen major locations of slag disposal. The bulk of the deposits are iron making (blast furnace) slags given the characteristic melilite (Ca-Al silicate)-dominated mineralogy. This is consistent with the age of deposits which are predominantly from the early to mid-20th Century, given blast furnace slags have been widely repurposed in recent decades. The presence of a range of secondary mineral phases (e.g. calcite) indicates that most sites have experienced extensive weathering. Slag deposits generally have low concentrations of trace elements, with modest enrichment of Cr and V at a small number of locations. Leaching tests demonstrated very low (i.e. mean concentration below 0.01 mg/L) leaching of most potential contaminants of concern (e.g. Cr, V, Zn) under saline conditions given the pH buffering offered by seawater. For Cr and V, this low leaching risk is consistent with the predominant trivalent form in the sampled slags observed with XANES. Only Mn and Zn show modest enrichment in leaching products under saline conditions, the former in concentrations that could be of environmental significance. However, given the large dilution capacity apparent in coastal disposal settings, it is likely that overall environmental risk is minimal given the predominant low leaching rates which are consistent with other studies. Furthermore, disposal of legacy wastes from an era of lax environmental regulation may be providing a range of potential broader benefits in terms of habitat creation and coastal defence provision that warrant further study, although there appear to be physical stability issues at some sites situated on high-energy coastlines. The use of modern iron and steelmaking slags for coastal land reclamation and restoration is already commonly practised internationally, for example in Japan (Nishijima et al. 2015; Okuda et al. 2014) and South Korea (Park et al. 2022), with examples of dumped slag deposits in coastal areas of Spain (Elorza and Recio 2023). Therefore, the assessment of their environmental behaviour in coastal settings presented here allows insight into potential leaching behaviours in global settings which can feed into future coastal regeneration efforts and better understanding of potential environmental risks.

### Supplementary information

ESM 1 (DOCX 1.44 MB)
