# Supplementary material for: Environmental behaviour of iron and steel slags in coastal settings
Source: Environ Sci Pollut Res Int. 2024 Jun 14;31(29):42428–44. doi: 10.1007/s11356-024-33897-4 (PMC11219386; doi:10.1007/s11356-024-33897-4)
Supplement: Supplementary file 1 — (DOCX 1.44 MB) [file 11356_2024_33897_MOESM1_ESM.docx]

**SUPPORTING INFORMATION**


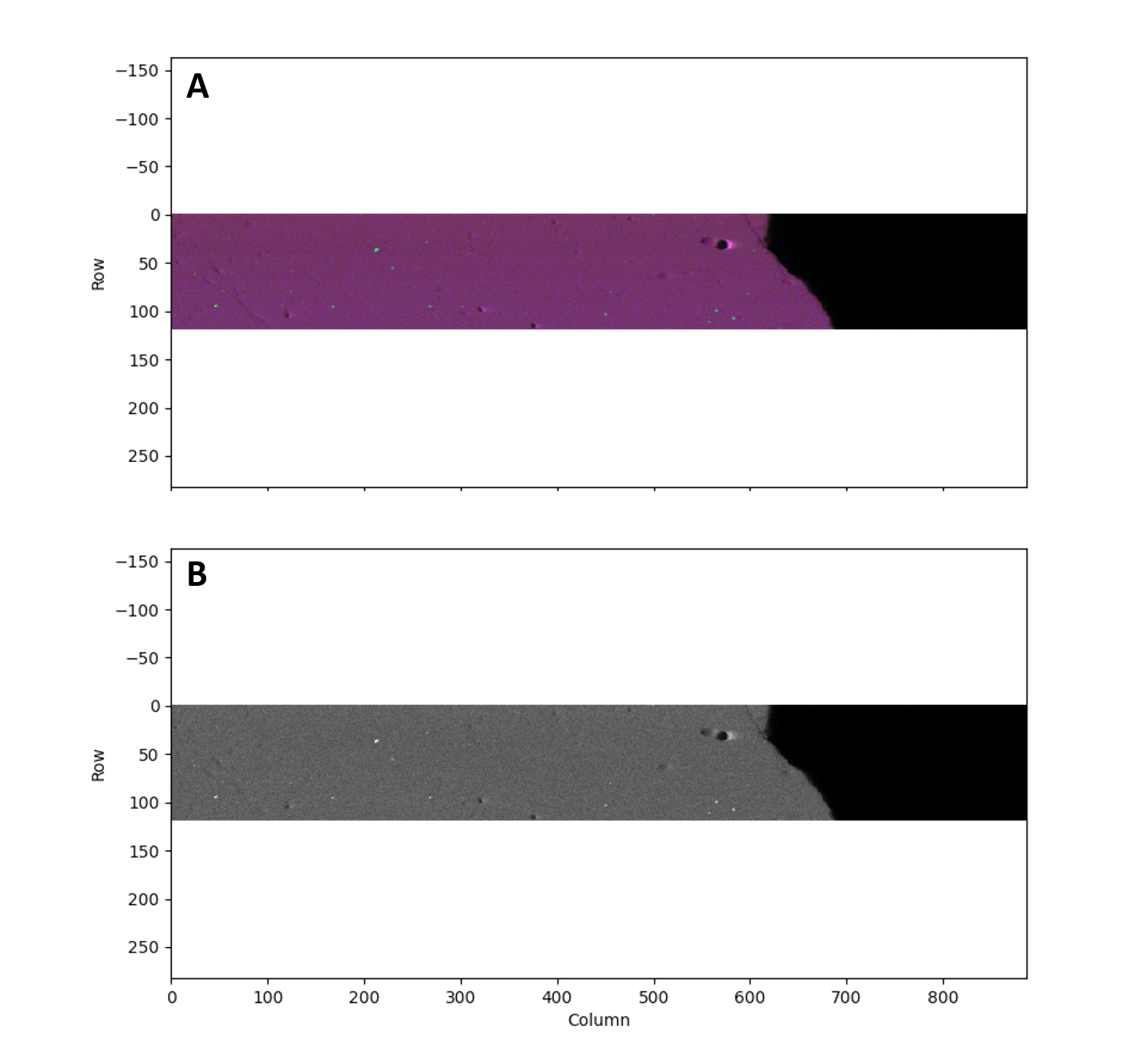


**SI Figure 1:** µXRF-generated elemental maps of ULV sample showing; A) hotspots of Cr (red = Ca, blue = Si, green = Cr), and B) hotspots of V (white spots). Axes values are pixels, with each pixel representing a 5x5 µm area on the sample.


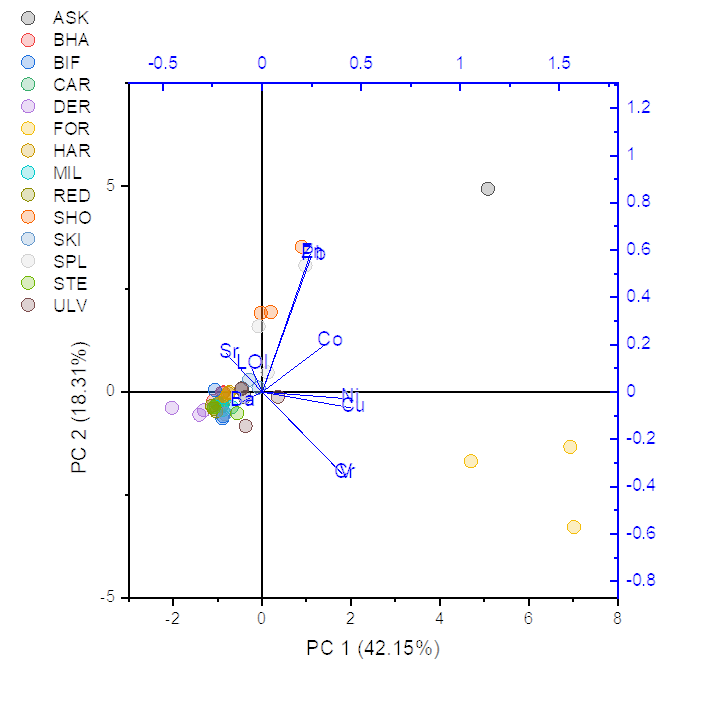


**SI Figure 2**: Principal component analysis (PCA) of slag composition by site.


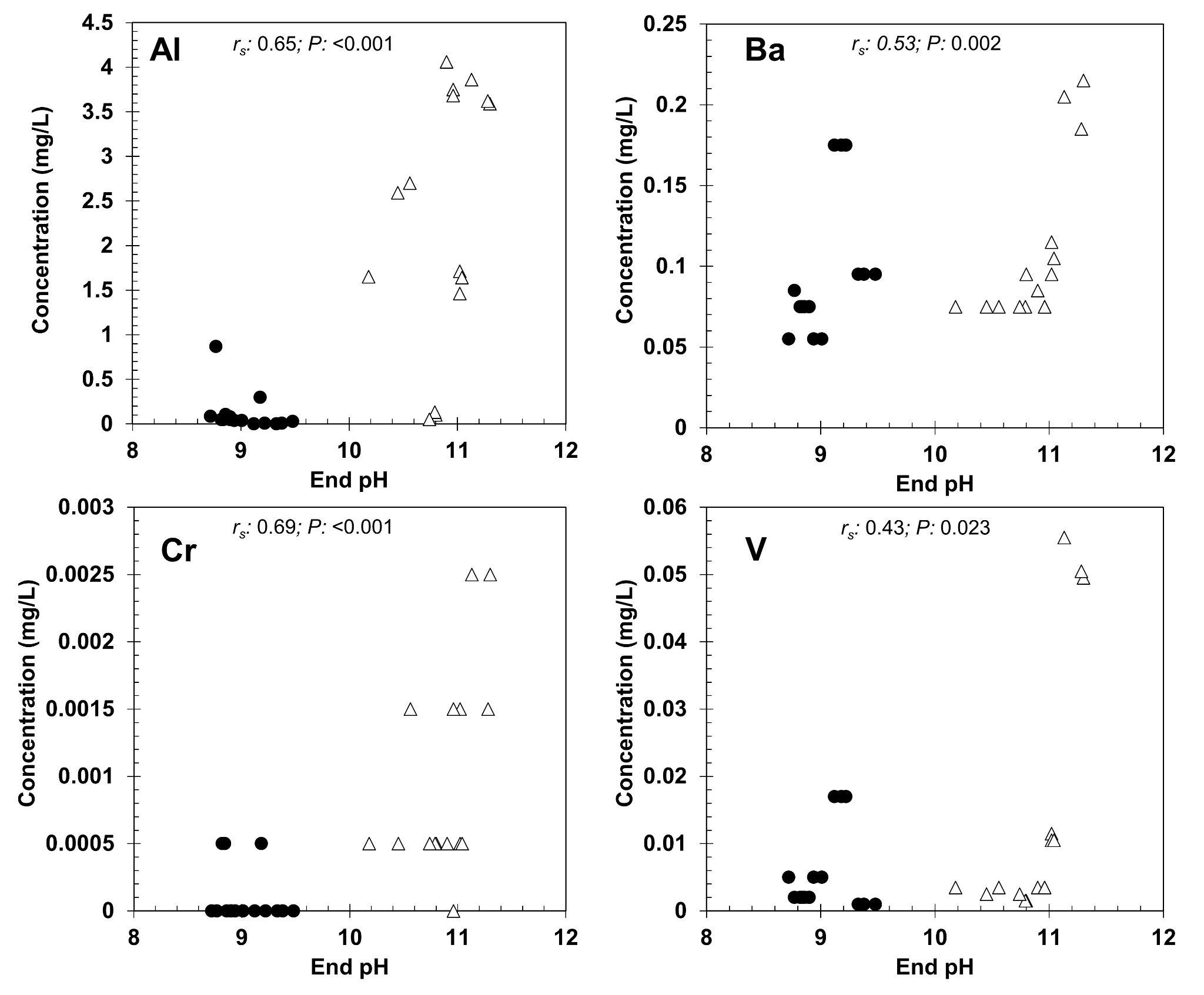


**SI Figure 3**: Relationship between final leaching test pH and selected elements. Closed circles show seawater treatments, open triangles show deionised water treatments. Spearman correlation coefficient and P value shown on each plot (degrees of freedom: 28 for each).


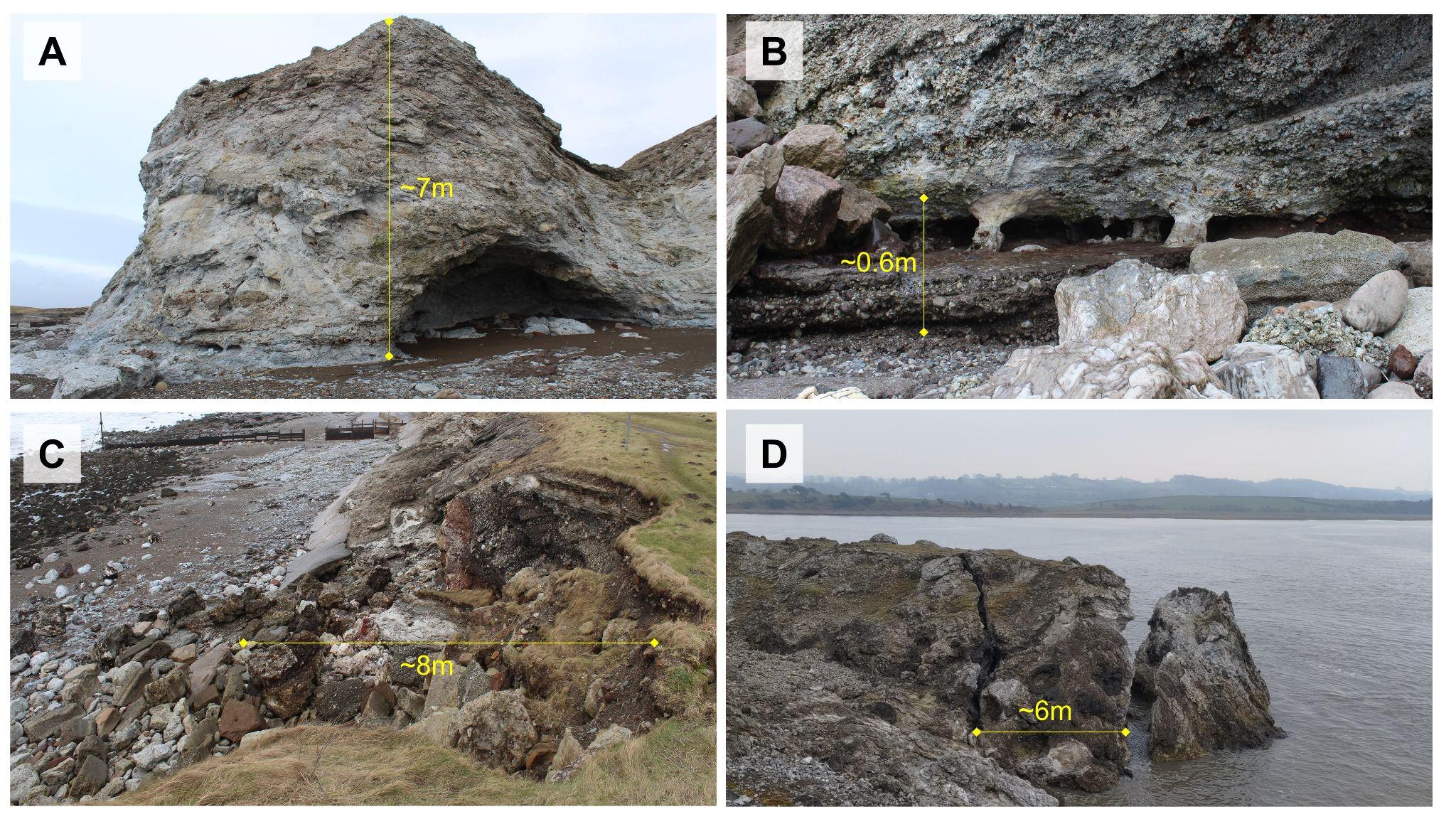


**SI Figure 4**: Example site photos showing: A: Major erosional feature developing in weathered slag at Harrington, Cumbria; B: Wave-cut platform and notch in slag at Derwent Howe, Cumbria (note also extensive secondary surface minerals); C: Erosion of slag cliff (and auxiliary concrete defence at base), Harrington, Cumbria; D: Tension crack and large block failure of slag bank at Carnforth, Lancashire, likely due to undermining of tidal flat deposits.

**SI Table 1**: SEM-EDS spot analysis used to identify phases present in XRD analysis and false-colour elemental maps in **Figure 4**. Elements present are shown as Atomic% and have been normalised to 100%.

| **Element**  (Atomic %) | **Melilite**  (Ca,Na)_2_(Al,Mg)[(Al,Si)SiO_7_] | **Larnite**  Ca_2_SiO_4_ | **Ca-Sulfide**  CaS | **CSiH**  Ca-Si-Hydrate | **Calcite**  CaCO_3_ | **Thaumasite**  Ca_3_Si(OH)_6_(CO_3_)(SO_4_).12H_2_O |
| --- | --- | --- | --- | --- | --- | --- |
| **O** | 57.7 ∓ 0.4 | 58.6 ∓ 0.6 | 27.9 ∓ 4.7 | 68.9 | 72.5 ∓ 1.0 | 70.9 ∓ 1.1 |
| **Na** | 0.7 ∓ 0.1 | n.d. | n.d. | 0.2 | 0.3 ∓ 0.1 | 0.1 ∓ 0.2 |
| **Mg** | 2.9 ∓ 0.5 | 0.1 ∓ 0.02 | n.d. | 2.63 | 0.2 ∓ 0.4 | 0.1 ∓ 0.1 |
| **Al** | 10.0 ∓ 0.9 | n.d. | 0.3 ∓ 0.1 | n.d. | n.d. | 0.2 ∓ 0.1 |
| **Si** | 12.5 ∓ 0.3 | 16.1 ∓ 1.0 | 1.9 ∓ 1.8 | 14.5 | 0.9 ∓ 1.6 | 5.8 ∓ 0.4 |
| **S** | 0.1 ∓ 0.02 | n.d. | 33.7 ∓ 5.1 | 0.7 | 0.3 ∓ 0.3 | 8.7 ∓ 0.5 |
| **Ca** | 16.5 ∓ 0.4 | 25.1 ∓ 1.7 | 35.9 ∓ 1.7 | 13.0 | 25.4 ∓ 2.6 | 13.7 ∓ 1.1 |
| **Ca/Si** | 1.3 | 1.6 | - | 0.9 | - | 2.4 |
| **Ca/S** | - | - | 1.1 | - | - | 1.6 |

N.d. = not detected; - = elemental ratio >10 (i.e. non-stoichiometric)
